# Bacterial contamination of forehead skin and surgical mask in aerosol-producing dental treatment

**DOI:** 10.1080/20002297.2021.1978731

**Published:** 2021-09-20

**Authors:** Madline P Gund, Gabor Boros, Matthias Hannig, Sigrid Thieme-Ruffing, Barbara Gärtner, Tilman R Rohrer, Arne Simon, Stefan Rupf

**Affiliations:** a Clinic Department of Operative Dentistry, Periodontology and Preventive Dentistry, Saarland University, Homburg, Germany; b Oral Surgery Clinic, German Armed Forces Central Hospital; Koblenz, Germany; c Institute of Medical Microbiology and Hygiene, Department of Hospital Hygiene, Saarland University, Homburg, Germany; d University Children’s Hospital, Saarland University Medical Center, Homburg, Germany; e Chair of Synoptic Dentistry, Saarland University, Homburg, Germany

**Keywords:** Aerosol, dental practice, infection control, maldi tof mass spectrometry, surgical mask, forehead skin

## Abstract

**Background:**

Bacterial contamination of dental professionals’ facial skin and protective equipment from treatment-related aerosols and droplets are poorly studied.

**Methods:**

This prospective study analyzed samples from 67 consecutive aerosol-producing dental treatments. Sterile nylon swabs served to collect samples from dental professionals’ foreheads before and after exposure. Contact samples were obtained from used surgical masks. Samples were incubated on agar under aerobic and anaerobic conditions. Bacteria were classified by MALDI-TOF mass spectrometry. We determined the frequencies of obligate and facultative oral bacteria and scored bacterial growth (0: none; 1: < 100 colonies; 2: >100 colonies; 3: dense).

**Results:**

Bacteria were detected in 95% of skin-swab and 76% of mask samples. Median bacterial scores were 2 for forehead samples before and after treatment, and 1 for masks. Obligate and facultative oral bacteria were more frequent (6% and 30%) in samples from exposed forehead skin, which also showed increased bacterial scores (28%). 5% of samples contained methicillin-sensitive *Staphylococcus aureus*; 3% contained obligate anaerobes.

**Conclusion:**

Exposed forehead skin was significantly less contaminated with obligate oral bacteria than expected based on surgical mask findings. Exposed forehead skin showed increased contamination attributable to aerosol-producing procedures. The forehead’s physiological skin microbiota may offer some protection against bacterial contamination.

## Introduction

Dental professionals are exposed to numerous potentially infectious factors [1] and personal protective equipment (PPE) is regularly contaminated during aerosol-producing dental treatment [2]. In this context, avoiding exposure of the airways, eyes and skin to potentially infectious agents, in particular bacteria and respiratory viruses, is of paramount importance [3]. However, microorganisms from patients’ oral cavities do not always present a risk to dental professionals. The risk of infection or outbreaks depends on microbial pathogenicity, the number of pathogens transmitted, and the exposed individual’s immune status [1,4]. Moreover, infectious agents can be transferred directly from patient to dental professional or vice versa, from patient to patient, or via chains of infection involving the staff, (hollow) instruments, clothing, or dental units [1,5–7].

In particular, treatments utilizing ultrasonic devices have been demonstrated to significantly contaminate the ambient air with bacteria [8]. Such aerosols may contain microorganisms from oral or dental unit biofilms, blood droplets, and blood-borne viruses [1,3,6,9–11], and may settle on the equipment, the members of the team, and their protective clothing [8,12].

The PPE for nonsurgical dental procedures consists of gloves [13], goggles, and surgical masks. In contrast to gloves, the correct use of surgical masks has not been a major issue so far. There are only few recommendations on the use of surgical masks in medicine and in dentistry [2,14,15] and very few systematic studies on their correct use. One study demonstrated that bacteria accumulate on the outer surface of the surgical mask during prolonged use for more than 2 h, a typical duration in many surgical disciplines [15]. In dentistry, however, the surgical mask is (1) usually worn for shorter periods of time, (2) frequently used in virtually every patient, and (3) regularly contaminated with microbial aerosols and patient’s saliva or blood during treatment. Aerosol-borne microorganisms from the oral cavity released during dental treatment survive on the outer surface of surgical masks [2].

Moreover, to the best of our knowledge, no previous study has investigated the bacterial load on dental professionals’ foreheads after performing aerosol-generating dental treatments. Considering that the working distance between the operator’s face and the treatment area is approximately 25–33 cm [16], there will inevitably be microbial contamination of the face, and protective clothing from aerosols and liquid splashes [16], because the dentist is within the zone of bacterial contamination during aerosol generating procedures [17].

The forehead is a body region that receives little attention in everyday clinical practice and is therefore frequently not protected by PPE. Yet in everyday clinical practice it is often repeatedly touched by the practitioners, wittingly or unwittingly, be it to wipe hair from their face or to remove water or aerosol splashes. This is often done unknowingly and without subsequently repeating hand hygiene. Thus, it is conceivable that germs may be transferred from the forehead to the environment. To support this hypothesis, it is first necessary to determine the extent to which the forehead is contaminated, particularly in comparison with surgical masks.

Against this background, we conducted the present study to investigate the potential bacterial contamination of the dental health care professional’s typically unprotected forehead and compared it with the external surface of the surgical mask worn during dental treatments.

## Materials and methods

### Setting

This prospective study was conducted at a university dental center. All instruments used for treatment were sterile, including handpieces and other items that potentially come into direct contact with the lips or oral cavity. The dental unit and the surrounding surfaces were routinely disinfected (Celtex® Wipes, Lotfex, Bremen, Germany; Incidin®, Dräger, Lübeck, Germany) in accordance with the pertinent recommendations of the German Commission of Hospital Hygiene and Infection Prevention (KRINKO). The room temperature was 20–22°C with 40–60% relative humidity.

### Subjects

Three fully trained dental professionals (two dentists and one dental hygienist) and 22 specifically instructed and supervised students in their 3rd or 4th year, here jointly referred to as ‘dental professionals’, participated as study subjects. During the aerosol-producing periodontal and restorative dental treatments they wore nonsterile, clean examination gloves (nitrile powder-free gloves: Abena, Zörbig, Germany), surgical masks (tie-band medical surgical mask type II, Mölnlycke Health Care, Düsseldorf, Germany), and protective eyewear (Safeview® eyewear, Halyard, Neunkirchen, Germany). Hand disinfection was performed before applying PPE. All study subjects were instructed not to touch the outer surface of their surgical mask during treatment.

### Patients

Patients without known infectious diseases were included in the study. No individual patient’s or professional’s data were recorded. All samples were anonymized. Verbal informed consent was obtained from all participants.

### Treatments

Typical dental treatments expectedly associated with small droplet and aerosol release were included: high/medium-speed preparation of tooth substances (n = 26) and periodontal treatments using ultrasonic devices (n = 41). The duration of treatment was 45–60 min. Droplets/aerosol suction was performed using a high-volume evacuation tube (8.0 mm in diameter; suction flow, 6.0 L/s) held by an assistant positioned on the contralateral side of the treated tooth, combined with a conventional dental suction cannula (3.3 mm in diameter; suction flow 1.1 L/s) placed lingually to the lower central incisors.

### Sampling

**Figure 1.**
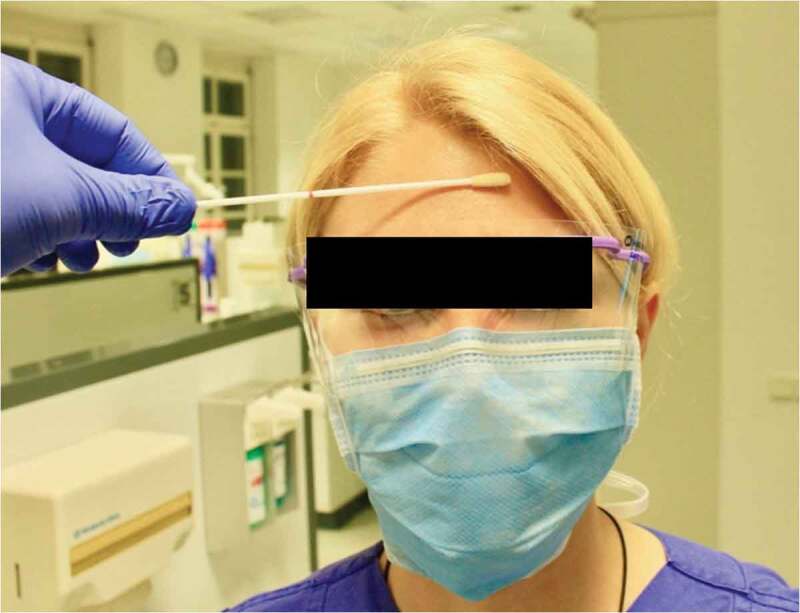
Sampling using an eSwab™. The entire forehead area not covered by hair was wiped off for 5–8 s

**Figure 2.**
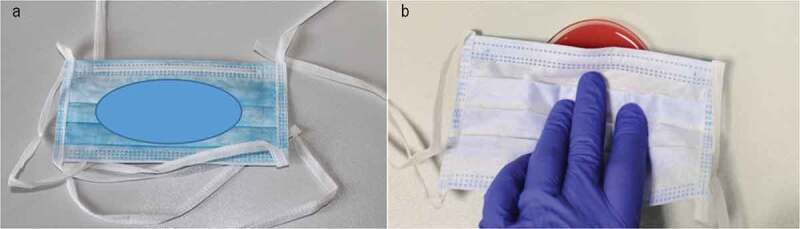
(a) Surgical mask with the area to be pressed onto agar indicated by the blue ellipse. (b) Surgical mask being pressed onto the agar surface

Microbiological sampling was conducted before and 60 min after starting dental work associated with the generation of small droplets/aerosols. Three samples were collected from each treatment session. Sampling involved bacterial swabs taken (1) from the forehead skin before treatment and (2) from the forehead skin 60 min after starting treatment (Figure 1), and (3) a contact sample from the used surgical mask (Figure 2a and b). The forehead was not cleaned or disinfected before taking the swabs. However, all participants were instructed to wash their faces with soap or shower gel at home in the morning as part of their personal hygiene. All samples were collected during the first treatment of the day between 9:30 and 11 a.m.

The forehead swab test was performed using the eSwab™ universal collection and transport system for aerobic and anaerobic bacteria (Hain Lifescience, Nehren, Germany), consisting of a tube with 1 ml of Amies medium and a nylon fiber flocked swab, which was moistened with sterile 0.9% NaCl solution (BD PosiFlush™, Becton Dickinson GmbH, Heidelberg, Germany) prior to swabbing.

Each surgical mask was pressed onto two different agar plates for 5 s each: BD Trypticase™ soy agar (TSA) plates (90 mm in diameter; Becton Dickinson) for aerobic cultivation, and BD™ Columbia Agar plates (90 mm in diameter) with 5% Sheep Blood (Becton Dickinson) for anaerobic cultivation. Five unused surgical masks served as controls and were processed as described above.

### Microbiology

The forehead skin swab samples were spread onto TSA and Columbia agar plates using the triple-streak plating method. To this end, bacteria were taken from the bacterial suspension using an inoculation loop and the first zig-zag streak was made on the agar plate. Bacterial density was then reduced by passing a second sterile inoculation loop through the first streak. This procedure was repeated with a third sterile inoculation loop to further reduce bacterial density, making it easier to isolate different species in the subsequent analysis. Agar plates were incubated at 36 ± 2°C for 48 h.

### Quantitative bacterial analysis

For each sample, bacterial colony counts on the agar plates were scored using a 4-grade scale: 0 = no bacterial growth; 1 = ≤10^2^ scattered colonies; 2 = > 10^2^ countable colonies; and 3 = dense bacterial growth with uncountable CFUs.

### Qualitative bacterial analysis

Phenotypically different colonies grown on the two different agar plates were classified using matrix-assisted laser desorption/ionization time-of-flight mass spectrometry (MALDI-TOF MS; Microflex® LT/SH, Bruker Daltonik, Bremen, Germany) and the flexControl and MALDI Biotyper® Compass software packages (Bruker Daltonik). Colonies were transferred to a stainless-steel target (96-spot target, Bruker Daltonik) using a toothpick, and overlayed with 2 μL of matrix (alpha-cyano-4-hydroxycinnamic acid, 20 mg/mL in 0.1% trifluoroacetic acid (TFA)/acetonitrile (1:2)). After crystallization, samples were washed twice with 0.1% TFA, and recrystallized in 0.1% TFA/acetonitrile (1:2). Measurements were carried out in a linear positive mode (delay 400 ns, voltage 20 kV, mass range 2–20 kDa, 240 laser shots per spot). The spectra were externally calibrated using the standard calibrant mixture, Protein Calibration Standard I (Bruker Daltonik). Measurements were continued until the bacterium was clearly identified. If a spectrum could not be assigned to a known species, it was classed as ‘unidentified’.

A change in the bacterial microbiota of the skin of the forehead was assumed under the following conditions: (I) detection of obligate oral bacterial species on the forehead skin after treatment, (II) detection of facultative oral species on the forehead skin and mask after treatment without detection of these species before treatment, (III) facultative oral species detected on the forehead skin after treatment or on the surgical mask, but not before treatment, and (IV) facultative species detected on the forehead skin before and after treatment with increases in bacterial scores.

### Statistics

The detection frequency and bacterial scores of the paired samples from forehead skin before and after treatment and surgical masks were statistically analyzed with the Wilcoxon signed-rank test, with *p* < 0.05 indicating statistical significance.

## Results

### General results

Table 1 shows the absolute and relative numbers of positive and negative samples of forehead swabs and total surgical mask samples. Most of the forehead skin swabs (94% and 96%) and 79% of the surgical masks were found to contain bacteria. The forehead contamination before and after treatment was not found to be different.

**Table 1.** Quantitative analysis of bacterial contamination on forehead skin and surgical masks

Samples (n = 67)	Positive	%	Bacterial score (median)
Forehead before treatment	63	94	2
Forehead after treatment	64	95.5	2
Surgical mask after treatment	53	79	1

No statistically significant differences between positive samples and bacterial scores for the forehead skin before and after treatment; Wilcoxon signed-rank test, *p* < 0.05

Quantitative analysis. Samples of surgical masks and forehead skin with bacterial growth on agar plates in absolute numbers and percentages. Median bacterial scores: 0 = no bacterial growth; 1 = ≤10^2^ colonies; 2 = >10^2^ colonies; and 3 = dense bacterial growth.

### Bacterial species

All bacteria identified in this study are presented in Table 2. Obligate and facultative oral bacteria as well as species of skin flora, species from other regions of the human body, and environmental bacteria were also detected.

**Table 2.** Bacterial species detected in forehead skin swabs and surgical mask samples from dental professionals exposed to treatment-related aerosols and droplets

Microbial species	Resident skin microbiota	Facultative or obligate oral microbiota	Typical habitat	Forehead before exposure	Forehead after exposure	Surgical mask
*Acinetobacter lwoffi*	+	f	skin, oropharynx, perineum	1	1	1
*A. ursingii*	+	−	skin, oropharynx, perineum	2	0	0
*Bacillus* spp.	−	−	ubiquitous, environment, soil	3	1	2
*B. cereus*	−	−	ubiquitous, environment, soil	1	4	5
*B. flexus*	−	−	poultry manure, environment	0	0	1
*B. pumilus*	−	−	ubiquitous, environment, soil	2	1	0
*B. subtilis*	−	−	ubiquitous, environment, soil, water	0	0	1
*Clostridium* spp.	−	−	ubiquitous, digestive tract	0	0	1
*Corynebacterium* spp.	+	f	skin, mucous membranes	0	0	1
*Escherichia coli*	−	−	gut, feces	0	0	1
*Kocuria* spp.	−	−	soil	1	0	0
*K. rhizophila*	−	−	soil	2	2	0
*Leclercia adecarboxylata*	−	−	colon of warm-blooded animals	0	0	1
*Lactococcus lactis*	−	−	production of cheese, kefir, and soured milk	1	0	0
*Micrococcus* spp.	+	−	ubiquitous, skin, environment	0	0	1
*M. luteus*	+	−	air, meat and dairy products, skin	3	1	8
*Neisseria subflava*	−	o	upper airways	1	0	0
*Propionibacterium* spp.	+	−	skin, urogenital tract, gut	4	3	0
*P. acnes*	+	−	skin	5	2	0
*Pseudomonas stutzeri*	−	−	soil	2	0	0
*Rothia dentocariosa*	−	o	oral cavity	0	0	5
*Staphylococcus aureus* (MSSA)	+	f	skin, mucous membranes	3	3	4
*S. auricularis*	+	f	skin, oral cavity	1	0	0
*S. capitis*	+	f	skin, mucous membranes	27	28	15
*S*. coagulase negative	+	f	skin, mucous membranes, oral cavity	1	0	1
*S. epidermidis*	+	f	skin, mucous membranes	50	52	33
*S. haemolyticus*	+	−	skin (axilla), mucous membranes	0	0	1
*S. hominis*	+	f	skin, mucous membranes	4	1	3
*S. pettenkoferi*	+	−	skin, blood cultures	1	0	0
*S. saprophyticus*	−	−	urinary tract, vagina, rectum, beef and pork products	5	6	5
*S. schleiferi*	+	−	skin	0	0	1
*S. warneri*	+	−	skin	1	0	1
*Streptococcus* spp.	−	o	oral cavity, digestive tract, urogenital tract	0	1	0
*S*. alpha hemolytic	−	o	oral cavity, digestive tract, urogenital tract	0	1	2
*S. constellatus*	−	o	oral cavity, digestive tract, urogenital tract	0	0	2
*S. infantis*	−	o	oral cavity, throat, nasopharynx	0	0	1
*S. mitis*	−	o	oral cavity, throat, nasopharynx	2	0	3
*S. oralis*	−	o	oral cavity biofilms	0	1	2
*S. parasanguinis*	−	o	oral cavity biofilms	0	0	2
Gram-positive viridans streptococci	−	o	oral cavity biofilms, caries, upper airways, nose	1	0	0

Qualitative analysis. Bacterial species in alphabetical order detected on the forehead´s skin and masks of dental professionals. The second and third columns indicate whether the species is classified as part of the resident skin microbiota (+/−) and as facultative (f) or obligate (o) oral bacteria. The numbers indicate the detection frequencies of the species on forehead skin before and after performing treatment and on surgical masks (max. n = 67 each). Colonies identified on the upper taxonomic levels are indicated as spp. or as Gram-positive or Gram-negative rods, coagulase-negative staphylococci, or alpha hemolytic streptococci.

### Quantitative results for forehead samples and surgical masks

Obligate oral bacteria were found on the forehead skin in 3% before and in 6% after treatment. Those bacteria were detected in 25% of the samples from surgical masks. The difference of detection frequencies between skin swabs after treatment and surgical mask samples was found to be statistically significant (*p* = 0.001; Table 3).

**Table 3.** Samples from surgical masks and forehead swabs found to contain obligate or facultative oral bacteria

	(I) Obligate oral bacteria	(II) Facultative oral species detected on the forehead skin ***and*** surgical mask after treatment but not before treatment	(III) Facultative oral species on the forehead skin ***or*** surgical mask after treatment but not before treatment	(IV) Facultative oral species detected on the forehead skin ***before and after*** treatment with increases in bacterial scores
Number of samples	67	67	61, II excluded	61, II excluded
Forehead before treatment	2 (3%)	–	–	17 (28%)^d^
Forehead after treatment	4 (6%)	6 (9%)	13 (21%)^b^	
Surgical mask	17 (25%)^a^		16 (26%)^c^	–

^a^significant difference from forehead skin after treatment, *p* = 0.001

^b^significant difference from forehead skin after treatment, *p* = 0.001

^c^significant difference from forehead skin after treatment, *p* = 0.0004

^d^significant difference from forehead skin after treatment, *p* = 0.005, Wilcoxon signed-rank test, *p* < 0.05

Samples from surgical masks and forehead swabs found to contain obligate or facultative oral bacteria. Data are displayed as numbers, percentages (in parentheses), and maximum numbers of samples.

Nine percent of the cases displayed facultative oral species on the forehead skin swabs and mask samples after treatment but not in the swabs from the forehead skin before treatment.

Facultative oral species were detected on the forehead skin after treatment for 21% of the swabs or on the surgical masks for 26%, but not before treatment. Also, these differences were statistically significant (*p* ≤ 0.001). An increase in bacterial scores of facultative species occurred in 28% of cases (*p* = 0.005; Table 3).

### Surgical mask controls

No bacteria were found in the samples from the unused surgical masks that served as controls (n = 5).

## Discussion

To our knowledge, this is the first study confirming statistically significant changes in the microbiota found on dental professionals’ foreheads after performing aerosol-producing dental treatments. In addition, our data demonstrate that the dental operator’s forehead is less likely to become contaminated during dental treatment than is the outer surface of the surgical mask, despite their distance from the patient being very similar. Our observations support previous assumptions that the skin possesses a natural protective mechanism which via various pathways either prevents microbial repopulation or eliminates transient bacteria that do not normally populate the site [18].

While the risk of bacterial contamination of the forehead skin after dental treatment has not been described so far, the distribution of fluid splashes on a protective face shield has been studied [19]. However, these reports investigated only droplet distribution on the face shield and did not analyze the actual qualitative and quantitative bacterial colonization of the facial skin. The results showed the nose region and inner corner of the eye to be the main area of exposure. Another study found the highest level of contamination in the region of the operator’s right arm and the assistant’s left arm [20]. Currently, there are no existing recommendations for dental professionals as to how to clean or disinfect their facial skin to remove bacterial contamination acquired during treatment.

The contaminated forehead skin must also be considered as a host surface facilitating the transmission of microorganisms from the patient’s oral cavity, albeit with a lower likelihood of successful contamination than expected for the outer surface of the surgical mask. Further transmission of pathogens may occur manually if exposed individuals touch their foreheads after treatment. However, during the current COVID-19 pandemic, dental treatment has changed considerably in our observation. Complete protective equipment is worn during aerosol generating procedures. Thus, at least for the time being, the protection of the dentist’s facial skin is guaranteed. The findings of the present study should, however, be taken into consideration when developing post-corona recommendations for future pandemics. The protection of the forehead by a face shield seems advantageous and should be considered a general recommendation.

The microbiological methodology used in this study had the advantage that the cultivation on agar only detects viable bacteria. The use of nucleic acid-based methods would probably have led to a larger number of detected species. This would have demonstrated the potential of aerosols to transport bacteria, regardless of their viability. From the infectious disease perspective, however, only viable bacteria pose a potential risk to the dental staff. The agar used usually serves to detect the majority of fast-growing bacteria. Slow-growing species may have been underestimated. However, it was to be expected that readily cultivable and robust bacteria, in particular, would play a role since the resident microbiota would offer a certain degree of protection against invading bacteria. Additionally, bacteria that spread easily would also be at an advantage if further contamination were to occur from the forehead or mask onto surfaces, other regions of the body, or other individuals. The MALDI-TOF MS analysis we used was restricted to colonies that were identified as different phenotypes. This may potentially have resulted in underestimation of the bacterial spectrum on both foreheads and surgical masks. In our study, surgical masks were worn for 60 min. Published data suggest, as shown by our own preliminary tests, that in the absence of aerosol-releasing treatments, surgical masks were completely free of detectable bacteria. Hence, this potential limitation is irrelevant to the conclusions of our study.

Forehead contamination rates were significantly lower than surgical mask contamination rates, with both sites exhibiting a similar spectrum of bacterial contaminants. The majority of the bacterial species detected in our study were typical members of the skin or oral microbiomes. The most prevalent species in this study was *Staphylococcus epidermidis*, which was found on at least two thirds of the examined surgical masks and foreheads. Contamination with other *Staphylococcus* spp. was observed in one out of five masks and foreheads. *Micrococcus luteus, Rothia dentocariosa, Streptococcus oralis*, and various *Bacillus* spp. were each detected on more than ten masks and foreheads.

Certain bacterial contaminants observed in our study are of particular clinical significance. The coagulase-negative staphylococci, such as *S. epidermidis* or even *S. aureus*, are all potentially multi-resistant pathogens. The prevalence of *S. aureus* was low in this study, lower than reported by others [21]. Moreover, this pathogen was detected in only three surgical mask samples after exposure to dental aerosols and droplets. No additional pathogens were found on the study subjects’ foreheads after performing treatment. This may be due to patient selection as patients were only enrolled in this study if they reported not having any general disease. Moreover, the participating dentists and dental staff were informed of, and highly compliant with, the strict hygiene standards maintained at our dental center. In any event, *S. aureus* naturally represents a high-risk pathogen, for which this study has demonstrated a potential transmission path.

The most frequently isolated pathogen in this study was *S. epidermidis*. It was detected in fifty forehead swab and 32 surgical mask samples. This high detection rate is in line with results from other studies [2,22]. *S. epidermidis* is the most common member of the coagulase-negative staphylococci found on human epithelial surfaces and must be considered an important nosocomial pathogen [23].

The other detected oral and dermal bacteria, such as *Staphylococcus capitis, S. oralis, M. luteus* or *R. dentocariosa*, and others, are part of the commensal microbiota. These bacteria are not pathogenic in healthy individuals, but may cause disease in immunosuppressed or immunocompromised patients [24–27]. However, a patient’s health status and the risk factors causing a facultative pathogen to become pathogenic are not always clear. Therefore, it is reasonable and sensible to implement consistent compliance with regulations and recommendations for the prevention of nosocomial infections [28]. Important factors that determine infection and the clinical manifestation of disease in dental professionals include the frequency of exposure to a pathogen, and its virulence [1]. Consequently, consistent preventive behavior is of great importance since it is impossible in the dental practice to assess whether a patient is carrying an obligate or facultative pathogen that may be transferred at a dose high enough to harm a susceptible dental health care professional.

## Conclusions

After aerosol-producing dental treatments, the foreheads of the dental staff participating in the present study showed significantly lower contamination with bacterial species from aerosols and droplets of patients’ oral fluids compared with the outer surface of their surgical masks. We hypothesize that the physiological microbiota of the forehead skin may offer some degree of protection against contamination with other microorganisms, including bacterial pathogens. Nevertheless, the exposed areas of the dental operator’s facial skin should be considered a potential threat to dental professionals and a source of nosocomial transmission of microbes. Dental professionals therefore need to reduce facial skin exposure and avoid touching surgical masks during and after treatment. The general use of a face shield should also be taken into consideration.

## Data Availability

The dataset used and analyzed during the current study is available from the corresponding author upon reasonable request.
